# “Did you ever drink more?” A detailed description of pregnant women’s drinking patterns

**DOI:** 10.1186/s12889-016-3354-9

**Published:** 2016-08-02

**Authors:** Evelyne Muggli, Colleen O’Leary, Susan Donath, Francesca Orsini, Della Forster, Peter J. Anderson, Sharon Lewis, Catherine Nagle, Jeffrey M. Craig, Elizabeth Elliott, Jane Halliday

**Affiliations:** 1 Murdoch Childrens Research Institute, Parkville, 3052 VIC Australia; 2 Department of Paediatrics, The University of Melbourne, Parkville, 3052 VIC Australia; 3 Telethon Kids Institute, Perth, 6845 WA Australia; 4 Judith Lumley Centre, School of Nursing and Midwifery, SHE College, La Trobe University, Melbourne, 3000 VIC Australia; 5 The Royal Women’s Hospital, Parkville, 3052 VIC Australia; 6 Quality and Patient Safety Strategic Research Centre, Deakin University, Geelong, 3220 VIC Australia; 7 Women’s and Children’s Division, Western Health, St Albans, 3021 VIC Australia; 8 Paediatrics & Child Health, Children’s Hospital Westmead, The University of Sydney, Sydney, 2006 NSW Australia

**Keywords:** Pregnancy, Alcohol, Prevalence, Epidemiology, Socioeconomic factors, Risk factors, Predictors, Binge drinking, Unplanned pregnancy

## Abstract

**Background:**

This paper presents drinking patterns in a prospective study of a population-based cohort of 1570 pregnant women using a combination of dose and timing to give best estimates of prenatal alcohol exposure (PAE). Novel assessments include women’s special occasion drinking and alcohol use prior to pregnancy recognition.

**Methods:**

Information on up to nine types of alcoholic drink, with separate frequencies and volumes, including drinking on special occasions outside a ‘usual’ pattern, was collected for the periconceptional period and at four pregnancy time points. Weekly total and maximum alcohol consumption on any one occasion was calculated and categorised. Drinking patterns are described in the context of predictive maternal characteristics.

**Results:**

41.3 % of women did not drink during pregnancy, 27 % drank in first trimester only; most of whom stopped once they realised they were pregnant (87 %). When compared to women who abstained from alcohol when pregnant, those who drank in the first trimester only were more likely to have an unplanned pregnancy and not feel the effects of alcohol quickly. Almost a third of women continued to drink alcohol at some level throughout pregnancy (27 %), around half of whom never drank more than at low or moderate levels. When compared with abstainers and to women who only drank in trimester one, those who drank throughout pregnancy tended to be in their early to mid-thirties, smoke, have a higher income and educational attainment.

Overall, almost one in five women (18.5 %) binge drank prior to pregnancy recognition, a third of whom were identified with a question about ‘special occasion’ drinking. Women whose age at first intoxication was less than 18 years (the legal drinking age in Australia), were significantly more likely to drink in pregnancy and at binge levels prior to pregnancy recognition.

**Conclusions:**

We have identified characteristics of pregnant women who either abstain, drink until pregnancy awareness or drink throughout pregnancy. These may assist in targeting strategies to enhance adherence to an abstinence policy and ultimately allow for appropriate follow-up and interpretation of adverse child outcomes. Our methodology also produced important information to reduce misclassification of occasional binge drinking episodes and ensure clearly defined comparison groups.

## Background

Prenatal alcohol exposure (PAE) can result in Fetal Alcohol Spectrum Disorders (FASD), an umbrella term for a range of impairments, including learning difficulties, executive dysfunction, impaired speech, motor problems and behavioural issues [1]. It is now well accepted that heavy and chronic PAE affects brain development [2, 3] and there is evidence from current recent systematic reviews and meta analyses for detrimental associations between moderate PAE and child behaviour, binge drinking and cognition [4] and between heavy PAE and gross motor function [5]. Another recent study suggests that binge drinking, especially early in pregnancy, is correlated with hyperactivity and/or inattention [6]. However, the evidence for neurodevelopmental harms from low and infrequent alcohol use during pregnancy remain equivocal [7–12]. Consequently, Australian and international policies recommend that it is safest for women to completely refrain from drinking alcohol in the periconceptional period and throughout pregnancy [13, 14].

Despite clear evidence that primary prevention of FASD is possible if prenatal alcohol exposure is avoided, up to 80 % of women drink during pregnancy, many before pregnancy recognition [15–17]. Contributing substantially to this early drinking is the high frequency of unplanned pregnancies, at least 30 % [18]. Even if a woman stops drinking as soon as she discovers she is pregnant, she may have been drinking, perhaps binge drinking, in a critical period of embryogenesis. While not drinking is the safest option, these data show that many women of childbearing age do not abstain from alcohol simply because there is a chance they could become pregnant.

This paper presents the drinking patterns of pregnant women in a large cohort study (Asking Questions about Alcohol in Pregnancy, or AQUA) underway in Melbourne, Australia, using a composite method to assess patterns of alcohol consumption [19, 20]. AQUA is unique in that, after focus group testing [21], a question on special occasion drinking was included to allow for collection of information on drinking episodes which fall outside a ‘usual’ pattern. This question was to encourage disclosure of infrequent events where alcohol use was higher than normal. Maternal characteristics are examined for different pregnancy alcohol consumption patterns, with a view to providing information which assists in early recognition of women who may drink alcohol prior to pregnancy awareness and/or those who continue to drink throughout pregnancy.

## Methods

### Study population

Women attending antenatal clinics at seven public hospitals located in Melbourne, Australia, between July 2011 and July 2012 were provided with information about the study by specially trained research staff and invited to participate. Pregnant women were eligible for inclusion if their pregnancy was less than 19 weeks gestation, they were 16 years of age or older, had a singleton pregnancy and spoke and read English. Women interested in participating in the study were invited to complete a consent form and a first questionnaire. All eligible women were invited until April 2012, after which abstainers, if this information was volunteered at recruitment, were no longer offered participation because the target number of the abstainer group had been reached. Of all 4788 approached, 27 % declined participation, 3035 women consented, 2046 completed at least one questionnaire, 73 % of whom completed all three pregnancy questionnaires and were eligible for this analysis (*n* = 1570). Additional details of data collection, participation rates and characteristics of women lost-to-follow-up, are described elsewhere [19].

### Questionnaires

The questionnaires were developed following a comprehensive literature review of existing survey measures of alcohol consumption during pregnancy, and identification of potential confounding, modifying and mediating factors. Focus groups with women of childbearing age augmented development of a set of questions and a pictorial ‘drinks guide’ to sensitively elicit accurate information about the type and amount of alcohol used during pregnancy [21]. Particulars of the full range of variables collected in the study are provided in the protocol paper [19].

### Alcohol questions

Detailed information on the quantity and frequency of alcohol consumption for the three months pre-pregnancy and for each trimester was collected. For women who stopped drinking in the first trimester, information was gathered on when they stopped and women were classified as drinking only prior to pregnancy recognition (pre-aware) or drinking throughout first trimester (post-aware).

Women were provided with a pictorial drinks guide listing the most commonly-consumed types and volumes of alcoholic drinks including red and white wine, champagne, beer, cider, spirits, alcoholic sodas, pre-mixed spirits, port, sherry, and cocktails. Women were asked to use the drinks guide to identify what type of alcoholic drink(s) they usually consumed, with provision for up to five types of drink. For each beverage identified, women were asked how often they usually drank this type of alcohol (less than once per month, 1–2 days per month, 1–2 days per week, 3–4 days per week, 5 or more days per week) and how many drinks they usually consumed on each occasion (less than one drink, 1–2 drinks, 3–4 drinks, 5–6 drinks and 7 or more drinks). The next question asked women if there were any special occasions (or difficult times) when more than their usual amount of alcohol was consumed, the frequency of these occasions, the drink types, and number of drinks per occasion. If a woman reported consuming seven or more drinks on any occasion they were asked to provide the maximum number.

### Categorisation of maternal alcohol consumption: prenatal alcohol exposure (PAE)

The level of alcohol consumption was derived from the drinks choices reported by the women, after conversion to standard drinks and then grams of absolute alcohol (g AA) per week. One standard drink in Australia is equal to 10 g of AA. The ‘low’ category was classified as ≤ 70 g AA per week and no more than 20 g AA per occasion. The ‘moderate’ group included women drinking ≤70 g AA/week, but consuming more than 20 g and less than 50 g per occasion. The ‘high’ group included women drinking >70 g AA/week, but never more than 49 g/AA per occasion. Binge drinking was classified as the consumption of ≥50 g AA per occasion.

Responses to the special occasion drinking question at each time point were converted to g AA and the number of drinking occasions and whether these were binge episodes or not were recorded. Estimates from special occasions were then combined with those obtained from ‘usual’ alcohol consumption to calculate a new maximum weekly intake.

At each of the five time points, a code was assigned to the woman depending on her weekly alcohol consumption level (none, low, moderate or high). If the woman had one or more binge episode during that time, ‘binge’ replaced the code for weekly alcohol consumption for that time point. Three PAE categories were created for women who abstained in pregnancy: lifetime abstainers; those who did not drink in the three months before pregnancy and those who drank in the three months before pregnancy. A small number of women were not categorised into any of these groups because of their unusual drinking patterns (e.g. abstained in trimester 1, then drank some alcohol at various levels later in pregnancy).

Final categorisation of prenatal alcohol exposure (PAE) is presented using a three-tiered approach: PAE tier 1 consists of women who were abstinent in pregnancy (not including lifetime abstainers) and women who drank any alcohol while pregnant; PAE tier 2 further categorises this latter group of women into those who only drank in trimester one and those who drank throughout; PAE tier 3 defines PAE in seven categories: women who were abstinent in pregnancy but not lifetime abstainers (control group); women who drank in the first trimester (either at low, moderate/high or at binge levels pre-aware) and were abstinent in trimesters two and three; and women who drank throughout pregnancy (either at low or moderate/high levels in the first trimester or at binge levels pre-aware).

### Maternal variables

A wide range of maternal demographic variables was collected. [19] The variables and the classifications used in this paper are maternal age (<30, 30–34, ≥35 years), parity (0, ≥1), marital status (partnered/un-partnered), ethnicity (Caucasian (e.g. White Australian, UK, other European), Asian and other (e.g. mixed race), education (secondary, diploma/trade, university degree), gross household income per year (up to $40,000, $40–100,000, >$100,000 AUD), current financial situation (living comfortably, doing all right, just getting by, struggling), region of residence (metropolitan, rural), smoking (no, yes) and planned pregnancy (no, yes). Maternal report of height and pre-pregnancy weight were used to calculate body mass index (BMI). To gauge possible individual variation in alcohol metabolism, the women were asked if they felt the effects of alcohol very quickly, quickly, normally, slowly, or very slowly. Women were also asked about their drinking history, how old they were when they first started drinking regularly, if they had ever been intoxicated after drinking alcohol (defined as slurred speech, unsteady on their feet, or blurred vision) and the age when they first became intoxicated from drinking alcohol. Responses were categorised by age into <18 years and ≥18 years to reflect the legal drinking age in Australia.

### Analysis

Data were prepared and analysed in Stata v14 [22]. Codes were assigned for mutually exclusive PAE patterns according to the pre-defined levels, timing and patterns of the exposure(s). Percentages of binge episodes disclosed as part of usual drinking patterns and after inclusion of special occasion drinking information were calculated with 95 % binomial confidence intervals.

Estimates of the prevalence of PAE were based on recruitment of the first 1201 pregnant women, before most abstainers were excluded. The remaining analyses are based on the full cohort (*n* = 1570).

Pearson chi square testing was used to examine the relationship between early maternal age at onset of regular drinking, or first intoxication (+/− 18 years) and each PAE group.

Multivariate logistic regression was used to examine associations between maternal characteristics and pregnancy drinking patterns as compared to abstinent women (control). Unadjusted and adjusted odds ratios (controlling for all characteristics significantly related to any of the drinking patterns) were calculated. For predictor variables with more than two categories (maternal age, perceived alcohol effect, income, BMI and education), *p* values from likelihood ratio tests were used to evaluate the predictors.

## Results

### Effect of special occasion drinking on pre-conception and early pregnancy alcohol consumption (Table 1)

**Table 1:** Effect of a special occasion question on disclosure of binge episodes amongst 1570 women in the AQUA cohort

		Number	% of cohort (95 % CI)
Binge episodes disclosed as part of:	3 months prior to conception		
	Usual pattern	269	17.1 (15.3–19.1)
	Special occasion	327	20.8 (18.8–22.9)
	Total	596	37.9
	After conception, prior to pregnancy recognition^a^		
	Usual pattern	193	12.3 (10.7–14.2)
	Special occasion	97	6.2 (5.0–7.5)
	Total	290	18.5

^a^Mean (SD) gestational age at pregnancy recognition: 4.9 (1.5) weeks

In the three months prior to conception, 269 of the 1570 women (17.1 %) declared a binge episode of drinking as part of their usual drinking. The special occasion question resulted in an additional 327 women reporting a binge episode, raising the total to 596 women (37.9 %) having consumed alcohol at binge levels prior to conception.

After conception but before pregnancy recognition, 193 of 1570 women (12.3 %) declared binge episodes as part of their usual drinking; another 6.2 % were identified with the special occasion question, resulting in almost one in five women (18.5 %) having binged prior to pregnancy recognition.

Following pregnancy recognition, five women declared a binge episode in trimester one, five women in trimester two and one in trimester three.

### Prevalence of PAE in study population (Fig. 1)

Overall, 496 (41 %) of pregnant women did not drink during pregnancy, 322 (27 %) drank in the first trimester only and another 329 (27 %) continued to drink throughout pregnancy. A small number of women drank in the second and/or third trimester only or reported an irregular pattern not able to be classified (5 %, *n* = 54).

Of those who drank only in the first trimester, 87 % stopped once they realised they were pregnant (*n* = 280). The 329 women who continued to drink throughout pregnancy did so at low to moderate levels, 39 % of them drinking at binge levels in trimester one (*n* = 128), and 53 % drinking at low or moderate levels in trimester one (*n* = 174).

### Alcohol consumption in the three months before pregnancy and association with PAE (Table 2)

PAE tier 1 showed that most women who did not drink in the three months prior to pregnancy, remained abstinent while pregnant (90 %). The second PAE tier revealed that the majority of women who drank at high or binge levels before pregnancy drank throughout pregnancy (62 and 50 % respectively). PAE tier 3 showed a large percentage of women continuing to drink at the pre-pregnancy level, regardless of whether they ceased alcohol consumption in trimester one or continued throughout (e.g. 52 % of low pre-pregnancy drinkers continued to drink at low levels, 43 % of moderate pre-pregnancy drinkers and 64 % of high pre-pregnancy drinkers continued at these levels). A high percentage of women who drank at binge levels prior to pregnancy had binge episodes before they found out they were pregnant (44 %).

### Age at drinking regularly or age at first intoxication and PAE (Table 3)

Of the 1115 women who reported their age at onset of regular drinking, 56 % drank regularly before 18 years of age (*n* = 626). There were no discernible differences in age of onset of regular drinking between pregnancy abstinence or alcohol use (PAE tier 1) and whether this influenced women drinking in trimester one only or throughout pregnancy (PAE tier 2). PAE tier 3 analysis revealed that women who reported drinking regularly before the age of 18 years were more likely to have binge episodes early in pregnancy and to continue throughout, than women who began to drink at a later age. (17.6 % compared with 12.0 %, *p* = 0.02).

Of the 1205 who reported their age at first intoxication, 54 % of women reported this occurring before 18 years of age (*n* = 651). This group of women was more likely to drink alcohol while pregnant (PAE tier 1: (74.8 % compared with 61.9 %)) and if drinking, more likely to drink alcohol throughout pregnancy (PAE tier 2: 44.7 % compared with 28.3 %, *p* < 0.001). Being underage at first intoxication also predicted early pregnancy binge drinking, especially in those who drank throughout pregnancy (PAE tier 3: 19.5 % compared with 6.5 %, *p* < 0.001).

### Maternal characteristics of women in each PAE group

Table 4 contains the results of the adjusted logistic regression analyses of nine maternal characteristics across PAE tiers 1 and 2. Table 5 shows the same analysis for the expanded, PAE tier 3 categories. Current financial situation, relationship status, maternal age and region of residence are not included as either no meaningful differences in the univariate analysis were noted between groups or group numbers were too small.

**Table 2:** Pregnancy alcohol exposure patterns three months before pregnancy and during pregnancy

	Alcohol drinking level in 3 months prior to pregnancy^a^					
	None	Low	Moderate	High	Binge	
	*N* (%)	*N* (%)	*N* (%)	*N* (%)	*N* (%)	Total *N* (%)
PAE tier 1						
Abstinent throughout pregnancy	261 (90.0)	82 (41.6)	79 (27.4)	3 (6.4)	69 (12.3)	494 (35.7)
Drank alcohol while pregnant	29 (10.0)	115 (58.4)	209 (72.6)	44 (93.6)	494 (87.7)	891 (64.3)
Total	290 (100.0)	197 (100.0)	288 (100.0)	47 (100.0)	563 (100.0)	1385 (100.0)
PAE tier 2						
Abstinent throughout pregnancy	261 (90.0)	82 (41.6)	79 (27.4)	3 (6.4)	69 (12.3)	494 (35.7)
Drank alcohol in trimester one only	22 (7.6)	64 (32.5)	119 (41.3)	15 (32.9)	210 (37.3)	430 (31.1)
Drank alcohol throughout pregnancy	7 (2.4)	51 (25.9)	90 (31.6)	29 (61.7)	284 (50.4)	461 (33.3)
Total	290 (100.0)	197 (100.0)	288 (100.0)	47 (100.0)	563 (100.0)	1385 (100.0)
PAE tier 3						
Abstinent throughout pregnancy	261 (90.0)	82 (41.6)	79 (27.4)	3 (6.4)	69 (12.3)	494 (35.7)
Low in T1, abstinent in T2 and T3	12 (4.4)	56 (28.4)	33 (11.5)	5 (10.6)	36 (6.4)	142 (10.3)
Moderate/high in T1, abstinent in T2 and T3	6 (2.1)	5 (2.5)	72 (25.0)	9 (19.1)	78 (13.8)	170 (12.2)
Binge pre-aware, abstinent in T2 and T3	4 (1.4)	3 (1.5)	14 (4.9)	1 (2.1)	96 (17.0)	118 (8.5)
Low in T1, low/moderate in T2 and/or T3	4 (1.4)	46 (23.3)	31 (10.8)	4 (8.5)	27 (4.8)	112 (8.1)
Moderate/high in T1, any level in T2 and/or T3	1 (0.3)	3 (1.5)	53 (18.4)	21 (44.7)	107 (19.0)	185 (13.4)
Binge pre-aware, low/moderate in T2 and/or T3	2 (0.7)	2 (1.0)	6 (2.1)	4 (8.5)	150 (26.6)	164 (11.8)
Total	290 (100.0)	197 (100.0)	288 (100.0)	47 (100.0)	563 (100.0)	1385 (100.0)

^a^Total excludes lifetime abstainers (*n* = 112) and 73 with irregular patterns, final *n* = 1385. Due to low numbers in the high level PAE, this category was combined with moderate PAE

T1, T2, T3: Trimesters 1, 2 and 3

**Table 3:** Relationship between early maternal age at onset of drinking and first intoxication and pregnancy alcohol consumption patterns

	Age at drinking regularly^a^			Age first intoxicated^b^		
	> = 18 yrs	<18 yrs	p chi^2c^	> = 18 yrs	<18 yrs	P chi^2c^
Total number of participants	626 (%)	489 (%)		554 (%)	651 (%)	
Abstinent throughout pregnancy	181 (28.9)	133 (27.2)	Reference	211 (38.1)	164 (25.2)	Reference
PAE tier 1						
Drank alcohol while pregnant	445 (71.1)	356 (72.8)	0.53	343 (61.9)	487 (74.8)	<0.001
PAE tier 2						
Drank alcohol in trimester one only	220 (35.1)	150 (30.7)	0.63	186 (33.6)	196 (30.1)	0.04
Drank alcohol throughout pregnancy	225 (35.9)	206 (42.1)	0.14	157 (28.3)	291 (44.7)	<0.001
PAE tier 3						
Low in T1, abstinent in T2 and T3	68 (10.9)	37 (7.6)	0.20	59 (10.6)	52 (8.0)	0.56
Moderate/high in T1, abstinent in T2 and T3	86 (13.7)	68 (13.9)	0.71	76 (13.7)	78 (12.0)	0.15
Binge preaware, abstinent in T2 and T3	66 (10.5)	45 (9.2)	0.74	51 (9.2)	66 (10.1)	0.02
Low in T1, low/moderate in T2 and/or T3	58 (9.3)	37 (7.6)	0.56	57 (10.3)	47 (7.2)	0.79
Moderate/high in T1, any level in T2 and/or T3	92 (14.7)	83 (17.0	0.28	64 (11.6)	117 (18.0)	<0.001
Binge pre-aware, low/moderate in T2 and/or T3	75 (12.0)	86 (17.6)	0.02	36 (6.5)	127 (19.5)	<0.001

^a^missing data on 270 (19.5 %)

^b^missing data on 180 (13.0 %). Approx 67 % of missing data was amongst the women abstaining in pregnancy

^c^Pearson chi square, *p* value

T1, T2, T3: Trimesters 1, 2 and 3

**Table 4:** Predictors of pregnancy alcohol use: Tiers 1 and 2

	PAE tier 1				PAE tier 2							
PAE	Any alcohol in pregnancy^a^				Abstinent after trimester 1^a^				Drank throughout^a^			
	*N*	AOR	95 % CI	*p* value	*N*	AOR	95 % CI	*p* value	*N*	AOR	95 % CI	*p* value
Participants	1318				873				911			
**Maternal age**												
<30 years	421	Reference			312	Reference			276	Reference		
30–34 years	539	1.40	(1.04–1.88)	0.03	327	1.13	(0.81–1.59)	0.47	388	**1.85**	**(1.29–2.66)**	**<0.01**
> = 35 years	358	1.28	(0.92–1.77)	0.14	234	1.18	(0.81–1.71)	0.39	247	1.37	(0.92–2.05)	0.12
**Pregnancy planning**												
Yes	1004	Reference			662	Reference			719	Reference		
No	314	**1.89**	**(1.40–2.55)**	**<0.001**	211	**2.05**	**(1.47–2.87)**	**<0.001**	192	1.60	(1.10–2.33)	0.01
**Parity**												
> = 1	689	Reference			438	Reference			506	Reference		
0	629	1.03	(0.80–1.33)	0.80	435	1.39	(1.04–1.86)	0.03	405	0.75	(0.55–1.03)	0.08
**Smoking**												
No	1091	Reference			750	Reference			751	Reference		
Yes	227	**1.94**	**(1.36–2.77)**	**<0.001**	123	1.33	(0.88–2.02)	0.17	160	**3.05**	**(2.00–4.67)**	**<0.001**
**Perceived alcohol effect**												
Normal	652	Reference			388	Reference			442	Reference		
Very/quickly	551	**0.47**	**(0.37–0.61)**	**<0.001**	409	**0.58**	**(0.44–0.78)**	**<0.001**	387	**0.38**	**(0.28–0.52)**	**<0.001**
Very/slowly	115	0.78	(0.50–1.20)	0.26	76	0.74	(0.44–1.24)	0.25	82	0.89	(0.52–1.53)	0.68
**Household income**												
<$40,000	168	Reference			131	Reference			109	Reference		
$40–100,000	578	1.16	(0.80–1.68)	0.44	394	0.93	(0.62–1.42)	0.75	410	1.53	(0.94–2.51)	0.09
>$100,000	522	**1.71**	**(1.15–2.54)**	**<0.01**	313	1.32	(0.84–2.05)	0.23	356	**2.43**	**(1.45–4.04)**	**<0.01**
Missing	50	0.96	(0.48–1.90)	0.91	35	0.75	(0.34–1.66)	0.48	36	1.57	(0.65–3.79)	0.31
**BMI**												
Normal	853	Reference			533	Reference			602	Reference		
Overweight	258	0.87	(0.63–1.18)	0.37	178	1.03	(0.72–1.47)	0.87	172	0.70	(0.48–1.03)	0.07
Obese	207	**0.53**	**(0.38–0.75)**	**<0.001**	162	0.76	(0.52–1.11)	0.16	137	**0.35**	**(0.22–0.54)**	**<0.001**
**Maternal education**												
Secondary	230	Reference			162	Reference			159	Reference		
Trade/Diploma	339	1.04	(0.72–1.50)	0.84	245	1.10	(0.73–1.67)	0.65	223	0.99	(0.62–1.58)	0.98
Tertiary	749	1.48	(1.04–2.11)	0.03	466	1.22	(0.81–1.84)	0.35	529	**1.88**	**(1.21–2.93)**	**<0.01**
**Ethnicity**												
White	1109	Reference			694	Reference			770	Reference		
Asian/other	209	**0.38**	**(0.27–0.52)**	**<0.001**	179	**0.61**	**(0.42–0.88)**	**<0.01**	141	**0.19**	**(0.12–0.30)**	**<0.001**

N: Sample size for multivariate analysis (i.e. number of cases with a complete set of predictors, except for income, where a ‘missing’ category was included because ~ 4 % of missing data)

AOR (95 % CI): Odds ratio and 95 % confidence interval adjusted for all predictors shown in table. Control group is abstinence in pregnancy, but not lifetime abstainer. Results are in bold font where a significant difference was found. For predictor variables with more than two categories (maternal age, perceived alcohol effect, income, BMI and education), *p* values from likelihood ratio tests (not shown) were used to evaluate the predictors. Likelihood ratio *p* values were <0.01 in all bolded results

^a^ **:** Control group is abstinence in pregnancy, but not lifetime abstainer

**Table 5:** Predictors of pregnancy alcohol use: Tier 3

	Drank in trimester 1, abstinent in trimester 2 and 3												Drank throughout											
PAE level	Low				Moderate/high				Binge pre-aware				Low in trimester 1				Moderate/high in trimester 1				Binge pre-aware			
	*N*	AOR	95 % CI	*p* value	N	AOR	95 % CI	*p* value	*N*	AOR	95 % CI	*p* value	*N*	AOR	95 % CI	*p* value	*N*	AOR	95 % CI	*p* value	*N*	AOR	95 % CI	*p* value
Participants	597				626				582				571				646				626			
**Maternal age**																								
<30 years	209	Reference			218	Reference			219	Reference			191	Reference			212	Reference			207	Reference		
30-34 years	228	1.11	(0.68-1.82)	0.67	242	1.46	(0.92–2.32)	0.11	209	0.90	(0.51–1.61)	0.73	229	1.62	(0.91–2.90)	0.10	247	1.51	(0.91–2.50)	0.11	264	**2.58**	**(1.54–4.31)**	**<0.001**
> = 35 years	160	1.23	(0.71–2.11)	0.46	166	1.33	(0.81–2.21)	0.26	154	0.95	(0.52–1.74)	0.88	151	1.19	(0.62–2.28)	0.60	187	1.58	(0.94–2.68)	0.09	155	1.16	(0.64–2.13)	0.62
**Pregnancy planning**																								
Yes	476	Reference			502	Reference			438	Reference			456	Reference			526	Reference			491	Reference		
No	121	1.67	(1.02–2.73)	0.04	124	1.32	(0.83–2.11)	0.25	144	**4.94**	**(2.98–8.20)**	**<0.001**	115	1.83	(1.05–3.20)	0.03	120	1.07	(0.63–1.81)	0.81	135	**2.00**	**(1.19–3.37)**	**<0.01**
**Parity**																								
> = 1	321	Reference			331	Reference			296	Reference			326	Reference			366	Reference			324	Reference		
0	276	1.21	(0.79–1.85)	0.37	295	1.33	(0.90–1.96)	0.15	286	**2.04**	**(1.25–3.33)**	**<0.01**	245	0.54	(0.33–0.90)	0.02	280	0.68	(0.45–1.04)	0.07	302	1.30	(0.84–2.03)	0.24
**Smoking**																								
No	533	Reference			538	Reference			499	Reference			502	Reference			550	Reference			519		Reference	
Yes	64	0.50	(0.23–1.13)	0.10	88	1.78	(1.05–3.00)	0.03	83	1.62	(0.88–2.98)	0.12	69	1.69	(0.81–3.52)	0.17	96	**3.62**	**(2.07–6.33)**	**<0.001**	107	**3.80**	**(2.20–6.55)**	**<0.001**
**Perceived alcohol effect**																								
Normal	236	Reference			258	Reference			250	Reference			224	Reference			291	Reference			283	Reference		
Very/quickly	309	0.75	(0.49–1.14)	0.17	314	0.64	(0.44–0.94)	0.02	276	**0.30**	**(0.18–0.50)**	**<0.001**	300	0.78	(0.49–1.24)	0.29	297	**0.33**	**(0.21–0.50)**	**<0.001**	280	**0.25**	**(0.15–0.40)**	**<0.001**
Very/slowly	52	0.62	(0.27–1.39)	0.26	54	0.68	(0.32–1.43)	0.31	56	0.90	(0.41–1.96)	0.78	47	0.48	(0.15–1.48)	0.20	58	0.96	(0.46–2.02)	0.92	63	1.41	(0.71–2.82)	0.33
**Household income**																								
<$40,000	94	Reference			93	Reference			88	Reference			84	Reference			87	Reference			82	Reference		
$40–100,000	279	0.71	(0.40–1.27)	0.25	297	1.08	(0.61–1.92)	0.79	270	0.91	(0.44–1.88)	0.81	271	1.10	(0.53–2.28)	0.79	303	1.43	(0.73–2.81)	0.30	288	2.59	(1.12–5.98)	0.03
>$100,000	197	0.88	(0.47–1.63)	0.67	211	1.46	(0.79–2.67)	0.23	199	1.75	(0.83–3.70)	0.14	193	1.56	(0.73–3.31)	0.25	228	1.89	(0.93–3.84)	0.08	229	**4.16**	**(1.77–9.77)**	**<0.01**
Missing	27	0.84	(0.29–2.40)	0.74	25	0.69	(0.21–2.29)	0.54	25	0.68	(0.18–2.63)	0.58	23	0.56	(0.11–2.90)	0.49	28	1.80	(0.57–5.66)	0.31	27	3.08	(0.84–11.27)	0.09
**BMI**																								
Norma	371	Reference			384	Reference			342	Reference			357	Reference			418	Reference			391	Reference		
Overweight	116	0.86	(0.51–1.46)	0.57	121	0.85	(0.52–1.40)	0.53	125	1.71	(0.99–2.95)	0.05	112	0.77	(0.43–1.37)	0.37	120	0.63	(0.38–1.05)	0.08	124	0.77	(0.45–1.33)	0.35
Obese	110	0.66	(0.36–1.19)	0.16	121	0.72	(0.44–1.20)	0.21	115	0.84	(0.45–1.59)	0.60	102	0.41	(0.20–0.86)	0.02	108	**0.28**	**(0.15–0.53)**	**<0.001**	111	**0.42**	**(0.23–0.79)**	**<0.01**
**Maternal education**																								
Secondary	111	Reference			123	Reference			110	Reference			104	Reference			117	Reference			120	Reference		
Trade/Diploma	154	0.81	(0.42–1.58)	0.54	178	1.03	(0.60–1.77)	0.93	171	1.93	(0.96–3.86)	0.06	148	1.07	(0.48–2.38)	0.87	168	1.06	(0.56–2.01)	0.85	165	0.86	(0.45–1.64)	0.64
Tertiary	332	1.45	(0.79–2.66)	0.23	325	0.89	(0.52–1.54)	0.68	301	1.54	(0.75–3.17)	0.24	319	2.48	(1.18–5.23)	0.02	361	**2.21**	**(1.21–4.04)**	**0.01**	341	1.42	(0.77–2.61)	0.26
**Ethnicity**																								
White	455	Reference			489	Reference			460	Reference			447	Reference			527	Reference			506	Reference		
Asian/other	142	0.82	(0.49–1.37)	0.45	137	0.65	(0.39–1.08)	0.10	122	**0.33**	**(0.16–0.71)**	**<0.01**	124	**0.36**	**(0.18–0.71)**	**<0.01**	119	**0.11**	**(0.05–0.25)**	**<0.001**	120	**0.14**	**(0.06–0.32)**	**<0.001**

***N*** **:** Sample size for multivariate analysis (i.e. number of cases with a complete set of predictors, except for income, where a ‘missing’ category was included because ~ 4 % of missing data)

AOR (95 % CI): Odds ratio and 95 % confidence interval adjusted for all predictors shown in table. Control group is abstinence in pregnancy, but not lifetime abstainer. Results are in bold font where a significant difference was found. For predictor variables with more than two categories (maternal age, perceived alcohol effect, income, BMI and education), *p* values from likelihood ratio tests (not shown) were used-evaluate the predictors. Likelihood ratio *p* values were <0.01 in all bolded results

Women who drank any alcohol while pregnant were more likely to have an unplanned pregnancy (PAE tier 1: OR 1.89 (95 % CI 1.40–2.55)), smoke (PAE tier 1: OR 1.94 (95 % CI 1.36–2.77)), have a household income of over AUD 100,000 (PAE tier 1: OR 1.71 (95 % CI 1.15–2.54)). They were less likely to perceive the effects of alcohol quickly (PAE tier 1: OR 0.47 (95 % CI 0.37–0.61)), be obese (PAE tier 1: OR 0.53 (95 % CI 0.38–0.75)) or of Asian/other ethnicity (PAE tier 1: OR 0.38 (95 % CI 0.27–0.52)). The PAE tier 2 comparisons revealed that pregnancy planning was only an important factor in those who ceased alcohol consumption in trimester one (PAE tier 2: OR 2.05 (95 % CI 1.47–2.87)), whereas smoking (PAE tier 2: OR 3.05 (95 % CI 2.00–4.67)), higher income (PAE tier 2: OR 2.43 (95 % CI 1.45–4.04)) and obesity (PAE tier 2: OR 0.35 (95 % CI 0.22–0.54)) remained associated only with women who drank throughout.

The PAE tier 3 analysis presented in Table 5 shows that the maternal predictors found to play a role in pregnancy drinking mostly related to binge drinking in the pre-aware period (regardless of whether they ceased alcohol consumption in trimester one or continued throughout) and to moderate/high dinking levels in women who drank throughout pregnancy. None of the maternal characteristics examined were associated with low or moderate/high drinking in those who drank only in trimester one and only Asian/other ethnicity was a protective factor for low level PAE throughout pregnancy.

## Discussion

This large population-based cohort of over 1500 women recruited in early pregnancy has provided data on the prevalence of the most common patterns of prenatal alcohol use, encompassing not only the critical period before pregnancy awareness, but also measures of pre-pregnancy drinking and drinking throughout pregnancy. A novel question about special occasion drinking in the measurement of PAE produced important information on binge drinking episodes which would have remained undisclosed in an otherwise best estimate of dose, timing and frequency of exposure [20]. The question was particularly valuable for alcohol use measured immediately prior to, or soon after conception. In this study, one in five women reported a binge episode in the time period before pregnancy awareness, a third of whom would not have been identified without the special occasion question.

Our tiered approach to the analysis of prenatal alcohol exposure patterns has identified a number of strong predictors able to be seen in the simplest of groupings (i.e. no alcohol compared with any alcohol), and allowed us to expose predictors of pregnancy drinking depending on timing (abstinent after trimester one compared with drinking throughout) or level of exposure (low, moderate/high or binge).

An important consideration in future health promotion programs is the finding that a substantial proportion of women who drink at binge levels in the three months before pregnancy also have binge episodes early in pregnancy. This is consistent with a review of 14 studies originating from the US, Europe, Australia, New Zealand, Japan and Uganda which found that pre-pregnancy drinking is one of the strongest predictors of drinking in pregnancy [23]. More recent studies [24–26] from Australia and New Zealand show similar correlations, adding that pre-pregnancy drinking also predicts alcohol consumption straight after birth at a time when safe breastfeeding should be encouraged [26]. Further to this, the results of our study add weight to the importance of delaying onset of regular alcohol use and intoxication to prevent binge drinking in adulthood [27]. We found that women who began drinking regularly and/or were first intoxicated before 18 years of age have a substantial risk of being a binge drinker in the first trimester.

This study has identified a number of predictors which may assist clinicians to determine whether a woman is possibly at risk of drinking alcohol while pregnant. Two of the strongest associations with binge drinking in the first trimester only, were having an unplanned pregnancy and no previous children. The high prevalence of alcohol consumption by women of childbearing age in many countries [28–30], coupled with the high proportion of unplanned pregnancies [18, 31–33] invariably results in many early unintentional alcohol exposures. Reassuringly, 87 % of the 27 % of women drinking only in the first trimester stopped when they became aware of their pregnancy, suggesting an understanding of the potential harms associated with alcohol in pregnancy. Similar cessation or reduction of alcohol consumption after pregnancy recognition has been described in other populations [26, 34, 35]. However, this early drinking is occurring at a critical time of fetal development (e.g. embryogenesis) and there need to be targeted health promotion strategies to cater for this distinct at risk population sub-group.

Pre-pregnancy behavioural counselling interventions have been trialled for women at risk of high drinking and a US Task Force Report on Fetal Alcohol Syndrome has shown many to be successful in increasing the likelihood of alcohol-free pregnancies through better use of effective contraception if not planning a pregnancy and/or a general reduction in alcohol use [36]. The challenge remains, however, to implement sustainable, multi-risk-level population-based prevention strategies in the primary care setting, aimed at all women of child bearing age [37].

A strong association between smoking and alcohol use in pregnancy in the Australian population has been demonstrated before [38, 39] and we have shown that women who smoke are at substantial risk of drinking at moderate to high or binge levels throughout pregnancy. This indicates that women who smoke may benefit from targeted alcohol education, even if no alcohol use is disclosed to their maternity clinician.

Of interest is the finding that higher household income (>$100,000 AUD per year) not only increased the likelihood of drinking throughout pregnancy but also predicted drinking at binge levels before the woman was aware of her pregnancy. Higher income has been noted by other studies as a predictor for ‘light social drinking’ [35], but not for binge drinking. Our finding may reflect the detailed information collected in this study about maternal drinking, which not only captured regular drinking patterns but also occasional binge drinking defined as having five or more standard drinks as a once off episode (≥50 g AA). When women are asked about their regular drinking patterns, they may not consider reporting a one-off occasion of binge drinking without being prompted for this information.

Women with a tertiary degree were twice more likely to continue drinking throughout pregnancy at moderate/high levels than women whose highest level of education was a secondary school certificate. While the evidence on the association of educational attainment on pregnancy drinking is conflicting, the Generation R study also reported that more highly educated women were also more likely to continue alcohol consumption in pregnancy [40]. Conversely, level of education was not associated with prenatal drinking in an analysis combining two US-based cohorts [41] and alcohol use was more common with lower education in two European studies investigating the effect of PAE with child outcomes [42, 43]. In Australia, the rationale for tertiary students using alcohol is well documented [44], with the proposition that engagement in a culture of drinking may lead to a tolerant attitude to alcohol consumption across the lifespan.

There were very few factors that reduced the likelihood of women drinking in pregnancy. One key protective factor was the woman’s interpretation of how she is affected by alcohol; women who reported experiencing the effects ‘quickly’ or ‘very quickly’ were less likely to drink in pregnancy. Greater awareness of individual variation in alcohol metabolism could be incorporated into messages, suggesting that regular drinkers who feel alcohol effects ‘normally’, may need to increase their vigilance in regards to contraception and pregnancy planning.

Other apparent protective factors identified were being of Asian ethnicity, most likely reflecting cultural, genetic and/or religious influences and a BMI greater than 30 (categorised as obese), perhaps due to general dietary guidance during pregnancy.

Interestingly, while our step-by-step approach revealed important factors, especially for likely binge level consumption and ongoing drinking, it did not identify any predictors of low level drinking, or even moderate/high levels for those who only drank in trimester one. These drinking patterns appear to simply be a reflection of women’s pre-pregnancy drinking and in the case of women who drink alcohol at low levels throughout pregnancy, this may be because she perceives these levels to be safe.

### Strengths and limitations of the study

A strength of this study is the accuracy of the alcohol measure [21] and a focus on the most frequent patterns of drinking, rather than heavy and chronic alcohol consumption. We believe this has played a key role in high participation and low attrition, but most importantly, in providing PAE data of the highest quality possible. Although our study measured PAE prospectively and thus optimised our ability to measure frequency, dose and timing of exposure, the use of self-reported questionnaires runs the risk of reporting bias. However, our focus group research showed that if questions on alcohol in pregnancy are appropriately contextualised and include an option to report unusual drinking episodes, this will encourage more accurate reporting, [21] a finding which appears confirmed with the high number of binge episodes reported in response to the special occasion question.

Our methodology also identified two groups of women who abstained from drinking alcohol in pregnancy (not including lifetime abstainers), those who drank alcohol in the three months prior to pregnancy and those who did not. While we combined the two groups for our analyses, it is likely that the exposure pattern of alcohol consumption in the three months prior to, but not during pregnancy, includes some women who inadvertently drank before pregnancy recognition and have self-reported incorrectly. On this basis we believe the best ‘control’ group in future examinations of child outcomes comprises women who reported no drinking in the three months before pregnancy. Further, detailed information on pregnancy alcohol consumption patterns, such as presented in this paper, should contribute to better confidence in prediction of outcomes after low to moderate PAE. Along with comprehensive reporting of alcohol consumption in each of the three pregnancy questionnaires, women provided information on drinking history and demographics, characteristics that are potentially associated with various exposure categories. Together, these may help target health promotion messages to those at greatest risk.

**Fig. 1:**
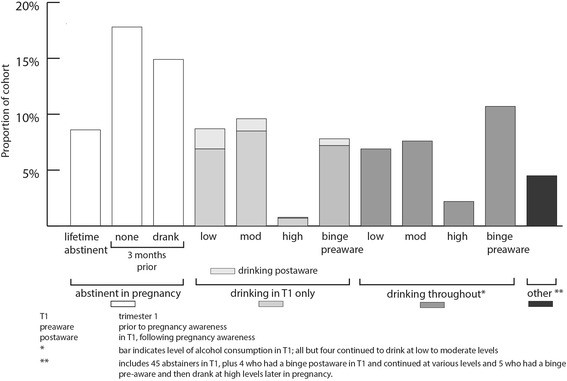
Prevalence of pregnancy alcohol exposure patterns - based on the first 1201 women recruited

The study included only English-speaking women from seven metropolitan public hospital antenatal clinics; therefore, women seeking care from private obstetricians, those from rural regions or those from culturally and linguistically different backgrounds may present with different patterns of drinking. However, the prevalence figures and predictors of PAE categories are specifically relevant to the target population which represents the majority of pregnant women in our State, where 70 % of women currently receive antenatal care in the public system. [45] Selection bias may have occurred if some women chose not to participate in the study because they did not drink alcohol while pregnant or because they were lifetime abstainers. Conversely, women drinking at higher levels may also have chosen not to participate or may have been more likely to drop out without completing all three pregnancy questionnaires needed to compute a PAE classification. All of this could affect our population prevalence estimates in Fig. 1. However, we achieved a sizeable number of women who abstained from alcohol (including lifetime abstainers), as well as a substantial number who drank throughout pregnancy, which we believe reflects realistic population drinking patterns.

## Conclusions

The collection of data on special occasion binge drinkers is a unique feature of this study. In addition to the implications of a special occasion question in the measurement of PAE, this finding is also of clinical relevance. Our data suggest that if maternity care professionals ask women about special occasion drinking, they may reveal important information on alcohol consumption, which may otherwise remain undisclosed. This information will allow the clinician to discuss the risks of alcohol in pregnancy and provide additional opportunities for referral.

The overarching message from this study is that two distinct populations of women exist: those who stop drinking as soon as they know they are pregnant and those who continue to drink, either because they are unaware of, or despite, the recommendation of abstinence. Some maternal characteristics may provide warning signs to health professionals who may be able to target a prevention message to women most likely to drink at binge levels. The first group are likely to have an unplanned pregnancy, not feel the effects of alcohol quickly and, if drinking at binge levels, have no previous children. Pregnancy planning and parity did not predict continued drinking beyond the first trimester. Rather, women in this category tended to have a higher education and income, smoke cigarettes and be in their early to mid-thirties.

The perceived alcohol effect (quickly or very quickly) is a new risk factor not described before and is likely to reflect a genetic marker of alcohol metabolism. It will be important to explore this potential individualised risk factor in the context of child outcomes and determine if it is a proxy measure for genetic susceptibility to fetal effects of alcohol.

Further, we conclude that having very detailed information on gestational timing, along with alcohol measures prior to and in very early pregnancy, as well as throughout pregnancy, will reduce misclassification through better identification of confounders, ensure a clearly defined control group and ultimately allow for appropriate interpretation of adverse child outcomes.
